# Musculoskeletal pain and its effect on daily activity and behaviour in Icelandic children and youths with juvenile idiopathic arthritis: a cross-sectional case-control study

**DOI:** 10.1186/s12969-022-00706-6

**Published:** 2022-07-15

**Authors:** Svanhildur Arna Oskarsdottir, Audur Kristjansdottir, Judith Amalia Gudmundsdottir, Solrun W. Kamban, Zinajda Alomerovic Licina, Drifa Bjork Gudmundsdottir, Bjorg Gudjonsdottir

**Affiliations:** 1 Department of Physical Therapy, School of Health Sciences, University of Iceland, Reykjavik, Iceland; 2 Children’s Medical Center, University Hospital of Iceland, Reykjavik, Iceland

**Keywords:** Children, Juvenile idiopathic arthritis (JIA), Musculoskeletal pain

## Abstract

**Background:**

Juvenile idiopathic arthritis is characterised by recurring episodes of acute inflammation, with joint swelling in one or more joints, often accompanied by pain. These episodes can now be controlled better than in the past because of a new category of medications. However, despite more stable disease activity, pain may continue to cause problems in the children with juvenile idiopathic arthritis and can reduce their performance of routine physical activities and participation in social or school activities.

**Aim:**

To evaluate the prevalence of pain, pain intensity, pain behaviour, and pain interference in Icelandic children with juvenile idiopathic arthritis compared with healthy peers.

**Methods:**

A cross-sectional, case-control study including 8-18 years old children; 28 with juvenile idiopathic arthritis and 36 in a control group. The children answered questions on pain experienced during the last 7 days, painful areas of the body and pain frequency. They completed short form versions of the Patient-Reported Outcome Measurement Information System (PROMIS) questionnaires on pain intensity, pain behaviour, and pain interference.

**Results:**

Significantly more children with juvenile idiopathic arthritis had pain compared with the control group (*p = 0.02*). Children with JIA also had a greater number of painful body areas (*p = 0.03),* more pain intensity (*p = 0.009*), and showed more pain behaviour (*p = 0.006*), and pain interference (*p = 0.002*). Children with juvenile idiopathic arthritis who had pain, experienced more pain interference (*p = 0.023*) than their peers who had pain. However, the groups did not differ in terms of pain intensity (*p = 0.102*) and pain behaviour (*p = 0.058*).

**Conclusion:**

The research results indicate that pain experience was different between children with juvenile idiopathic arthritis and the control group. The results suggest that further research of the role of pain management on functional outcomes in children with juvenile idiopathic arthritis is needed.

## Background

Juvenile idiopathic arthritis (JIA) is not a single disease but represents several subtypes with various clinical patterns of arthritis that begin before the age of 16, persist for longer than 6 weeks and are of unknown cause [1, 2]. JIA is characterised by recurring episodes of acute inflammation, with joint swelling in one or more joints, often accompanied by pain, sleep problems, fatigue, morning stiffness and difficulty performing activities at home and participating in school and other social activities. When the disease is inactive the symptoms are less prominent [1, 3].

Persistently active disease plays a major role in causing joint damage and physical functional disability [1, 3]. Evaluation of disease activity is a fundamental component of the clinical assessment of children with JIA. Juvenile Arthritis Disease Activity Score (JADAS) is commonly used to evaluate the disease activity in standard clinical care [4].

To ensure the best possible health outcomes, the management of a child with JIA should involve a team effort over a long period of time. The team includes the child and family who are involved in decision-making on all aspects of the health management and different health professionals depending on the child’s individual needs. The aims of intervention in JIA are to prevent joint damages, decrease the symptoms and increase the length of the remission periods and thereby maintain or improve the activity and social participation of children with JIA [5]. The intervention includes pharmacological management and non-pharmacological interventions by various health professionals [5].

Previous research has consistently demonstrated that pain is a common, clinically significant symptom in children with JIA [6, 7]. Chronic and recurrent pain that is not associated with diseases is also common in the general population of children and adolescents. A systematic review of studies between 1991 and 2009 demonstrated prevalence rates in children and adolescents that varied substantially. Musculoskeletal pain rate was in a range of 4-40% and was higher in girls and increased with age [8]. The difference between the general population and children with JIA is that the pain among the children with JIA is related to disease with recurring episodes of acute inflammation. These episodes can now be controlled better than in the past because of a new category of medications: biologic disease-modifying anti-rheumatic drugs [9].

Despite stable disease activity, pain continues to be a problem in children with JIA and may in some instances impair daily functioning and quality of life of the children [10–12]. In a study by Bromberg et al. [6], 59 children with JIA (ages 8–18 years) provided ratings of pain, stiffness, and fatigue intensity and functional limitations using a smartphone electronic diary three times each day for 1 month. The children reported moments of pain in 66% of the diary entries. No children reported being pain free during the reporting period. The pain that was reported was not related to the disease activity and joint inflammation. The children reported pain even though 79% of children were prescribed a disease-modifying antirheumatic drug and 47% were prescribed a biologic agent. The results indicate that effects of specific medications on pain are not straightforward [6]. Studies have explored factors influencing pain perception in children with JIA and indicate that central sensitization [2, 13, 14], psychological [15, 16], and environmental factors [17] may play a role in pain management.

The presence of pain impacts the lives of children with JIA, reducing their performance of routine physical activities and participation in social or school activities. The type of JIA, disease activity and disease severity contribute to the impact on activities and participation [5, 6, 18, 19]. A qualitative study of which the aim was to assess what clinical features, in the course of JIA, were most important for youths with JIA, their parents and clinicians showed that the core features most agreed on were the number of active joints, pain, and participant-defined quality of life [20]. A number of studies have demonstrated relationships between pain and activity limitations. Symptoms of pain, stiffness, and fatigue were significant predictors of restricted participation in school and social activities [19, 21]. Participation in school and in physical education was lowest when the children were newly diagnosed but increased during the disease course. School absence was related to disease activity and pain [19].

The aim of this study was to evaluate the prevalence of pain, the pain intensity, pain behaviour and pain interference in Icelandic children with JIA compared with their healthy peers.

## Materials and methods

### Design

A quantitative case-control study with cross-sectional design was used.

### Setting

This study took place in Iceland and was carried out as part of a larger study in collaboration with the paediatric rheumatology team at the National University Hospital of Iceland. 

### Participants and procedure

Children with JIA aged 8-18 years and their comparable peers were participants in the study. Children with diagnoses of all subtypes of JIA (except systemic onset) in The National University Hospital of Iceland’s medical record system from 2016 to 2019 were invited to participate. The control group was a random sample of children from the Registers Iceland. The inclusion criteria for both groups were residence in the southwestern part of the country including the capital area; being without disabilities; and both the children and parents having sufficient comprehension of Icelandic language. There were 48 eligible children with JIA. A list of 500 children was obtained from the Registers Iceland. Children who met the criteria were invited to participate (*n* = 249) in the control group. The children in the control group were paired with the group with JIA regarding to age and sex.

### Data collection

Data collection took place at the National University Hospital of Iceland, from October 2019 to March 2020. Information on subtypes of JIA and age of the group with JIA was obtained from the hospital medical record system, and on the age of the control group from the Registers Iceland. The legal guardians of the children with JIA were contacted by telephone and the study was presented to them. An introduction letter was then emailed to them, and the recipients were asked to reply if their children were willing to participate. If there was no response to the email, a researcher followed up with a phone call and encouraged participation.

The legal guardians of the children in the sample for the control group were texted a request to participate in the study, and they asked to reply if their children were willing to participate. A reminder notice was sent to non-respondents 1 week after the initial message. If no response had been made after several days, a researcher called the parents, encouraging them to participate. Snowball sampling was used in the last stage of data collection to obtain a large enough control group.

The children came to the hospital. The children with JIA underwent a medical examination by a paediatric rheumatologist, including an evaluation of the disease status with JADAS27. The height and weight of all the children were measured, and they answered a survey using the SurveyMonkey online survey tool.

### Outcome measures

The survey included questions on the child’s sex, pain experience and pain frequency during the last 7 days and location of painful body areas, and three short forms of the Patient-Reported Outcome Measurement Information System (PROMIS) questionnaires. The PROMIS measures were developed by the National Institutes of Health in the USA with the aim of assessing the physical, mental, and social health of individuals with chronic diseases. PROMIS instruments are administered as either fixed-length short forms or computer adaptive tests. Regardless of the administration method, each person’s score is placed on the same PROMIS metric [22].

Three short forms which all include questions on pain during the previous 7 days were used in this study.

PROMIS Paediatric Numeric Rating Scale v1.0 – Pain Intensity 1a includes one question on pain intensity. The children rated their pain during the last 7 days on a scale of 0 (no pain) to 10 (worst pain you can think of) [22].

PROMIS Paediatric Short Form v2.0 – Pain Interference 8a consists of eight questions on the consequences of pain on relevant aspects of one’s life. This includes the extent to which pain hinders engagement with social, cognitive, emotional, physical, and recreational activities [22].

PROMIS Paediatric Short Form v1.0 – Pain Behaviour 8a includes eight questions on behaviours that typically indicate to others that an individual is experiencing pain. These actions or reactions can be verbal or nonverbal, and involuntary or intentional [22].

The psychometric properties of the short PROMIS paediatric questionnaires have been studied in several studies, including children with JIA, demonstrating good content validity and high reliability [23–26].

### Ethics

The children and parents received verbal and written information and the parents signed an informed consent prior to participating in the study. Children over 12 years also signed the consent. Participation was voluntary and anonymous. Participants were informed about their right to withdraw from the study at any time without any consequences. Ethical approval for the study was granted by The National Bioethics Committee (VSN-19-141).

### Data analysis

The Microsoft Excel 2017 program (version 15.37) and the Jamovi statistics program (version 1.1.9.0, https://www.jamovi.org) were used to process the data.

Background information and the results of pain were described by mean and standard deviation for frequencies, and proportions for the categorical variables. The raw values for the Pain Interference and Pain Behaviour questionnaires were calculated and converted into T-scores with an interval scale using a calculator called HealthMeasures Scoring Service (https://www.assessmentcenter.net/ac_scoringservice). PROMIS scores have a mean of 50 and standard deviation (SD) of 10 in a reference population. The reference population is the population to which scores are compared to establish normative values (e.g., general population, clinical population). The reference population for the Pain Interference measure is healthy children in the USA and the reference population for the Pain Behaviour scale is children with chronic pain in the USA [27]. A higher T-score indicates more problems in both questionnaires [28]. There was missing data in the pain interference scale. A question of how hard it was for the children to run when they had pain was deleted from the online survey, by mistake. The HealthMeasures Scoring Service uses “Expected A Priori” pattern response scoring methods. These scoring methods use any available non-missing responses for an individual respondent to predict the most accurate score possible for any scores that are missing [28].

The chi-square test was used for a nominal scale data to determine whether the two groups were associated. When the data had normal distribution, an independent t-test was used to determine if there was a significant difference between the two groups. Kruskal-Wallis analysis of variance was used when the data was not normally distributed. The significance level was *p* ≤ 0.05 in the analyses.

## Results

**Fig. 1:**
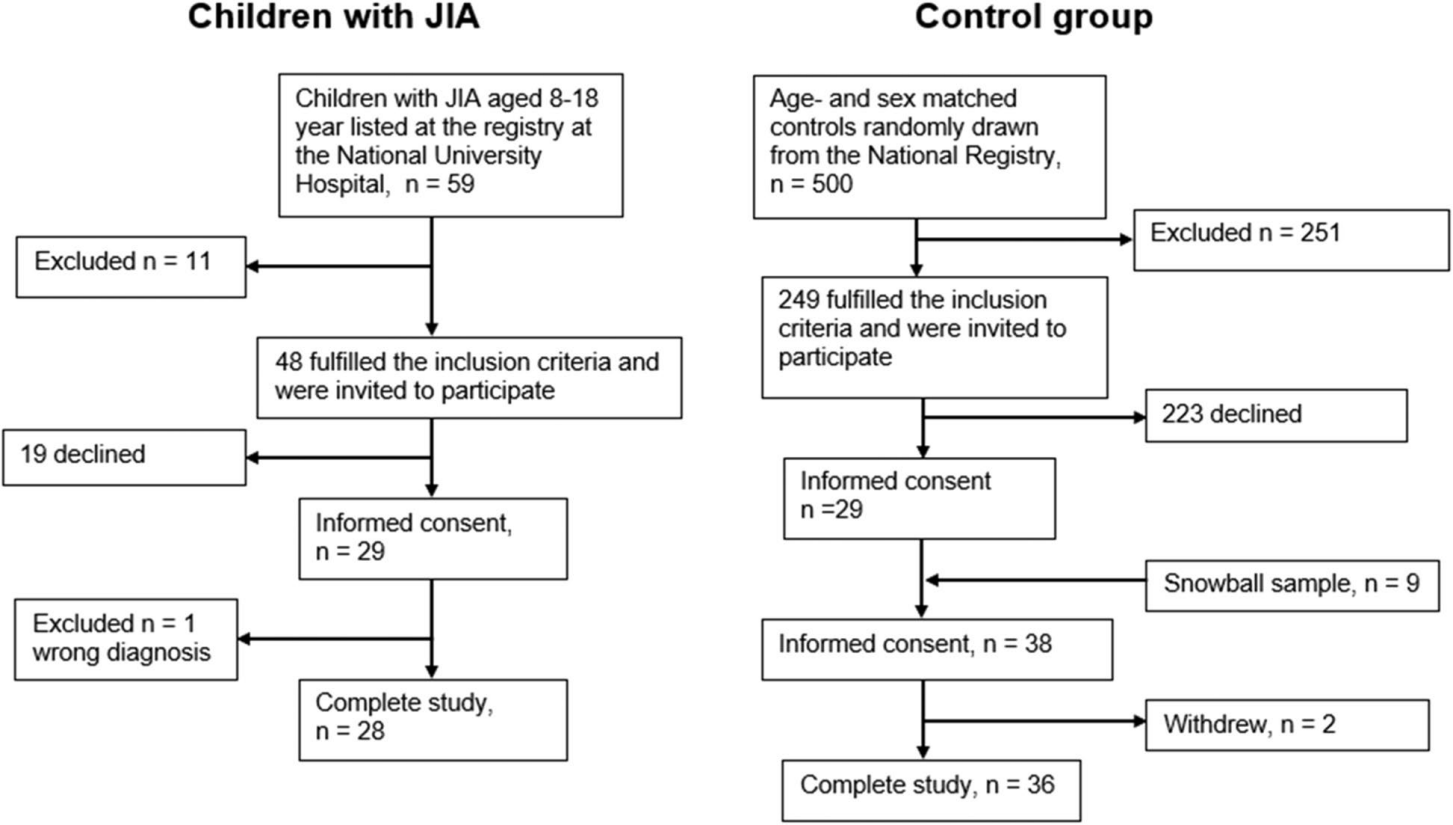
Flowchart of participant inclusion. JIA: juvenile idiopathic arthritis

A total of 28 children with JIA participated in the research, which was 58% of those who were invited. With the sample from the Registers Iceland and the snowball sample, 36 participants were in the control group (Fig. 1).

Characteristics of both groups, subtypes of JIA and JADAS27 scores are shown in Table 1. There was no difference between the groups in terms of sex, age, height, weight, and body mass index.

**Table 1:** Characteristics of children with juvenile idiopathic arthritis (JIA) and controls, and subgroup diagnoses of the children in the research group according to the diagnostic criteria of the International League of Association for Rheumatology. There was no difference between the groups in terms of sex, age, height, weight, and body mass index

	**Children with JIA (*n*** **= 28)**	**Control group (*n*** **= 36)**
Age in years, mean ± SD	12.9 ± 3	13.1 ± 3.1
Female, %	57	56
Height in cm, mean ± SD	156 **±** 13.8	159 ± 15.2
Weight in kg, mean ± SD	51.2 **±** 19.5	52.3 **±** 13.3
BMI in kg/m^2^, mean ± SD	20.2 **±** 4.6	20.3 **±** 2.7
JADAS from 0 to 57	2.9 **±** 3.1	
Children with joint inflammation, n	6	
	**Arthritis subtype n (%)**	
Oligoarthritis	13 (46.4)	
Rheumatoid factor positive polyarthritis	1 (3.6)	
Rheumatoid factor negative polyarthritis	2 (7.1)	
Enthesitis related arthritis	5 (17.9)	
Psoriatic arthritis	2 (7.1)	
Undifferentiated arthritis	5 (17.9)	

### Information about pain

Table 2 shows the results of questions about pain presence among the groups, pain frequency, and number of painful body areas during the last 7 days. Significantly more children with JIA experienced pain compared to the control group X^2^(1, *N* = 64) = 5.63, *p = 0.02*. Half (50%) of the children with JIA had recurrent pain, but only 25% of the control group.

**Table 2:** Number (n) and percentage among the groups for pain presence, frequency, painful body areas and the results from the Patient-Reported Outcome Measurement Information System (PROMIS) questionnaires (T scores and *p*-values) during the last seven days

**Single questions and questionnaires**	**Answer**	**JIA (*n*** **= 28)**	**Control group (*n*** **= 36)**	***p*-value**
Did you experience pain in the past 7 days?	Yes, n(%)	16 (57.1)	10 (27.1)	0.02
	No, n(%)	12 (42.9)	26 (72.2)	
How was the frequency of the pain in the past 7 days?	Persistent n(%)	1 (3.6)	0 (0)	
	Recurrent n(%)	14 (50)	9 (25)	
	Once n(%)	1 (3.6)	1 (2.8)	
	No pain n(%)	12 (42.9)	26 (72.2)	
Where was the pain?	More than one body part n(%)	10 (35.7)	2 (5.6)	0.03
	One body part n(%)	6 (21.4)	8 (22.2)	
	I did not experience pain n(%)	12 (42.9)	26 (72.2)	
		**JIA (*n*** **= 28)** mean ± SD	**Control group (*n*** **= 36)** mean ± SD	***p*-value**
PROMIS PAEDIATRIC PAIN INTENSITY (0-10)		3 ± 2.89	1.17 ± 2.04	0.009
PROMIS PAEDIATRIC PAIN BEHAVIOUR – SHORT FORM (T-score)		38.4 ± 11.3	31.1 ± 8.68	0.006
PROMIS PAEDIATRIC PAIN INTERFERENCE – SHORT FORM (T-score)		42.8 ± 8.97	36.6 ± 5.39	0.002

A chi-square test showed a significant difference between the two subgroups who had pain, in the number of painful areas of the body, as children with JIA had pain in more than one part of the body X^2^(1, *N* = 26) = 4.47, *p = 0.03.*

### PROMIS results

Table 2 shows the results of from the Patient-Reported Outcome Measurement Information System (PROMIS) questionnaires. Since the data were not normally distributed, a Kruskal-Wallis test was used to compare the results of the groups for each PROMIS questionnaire. The children with JIA had significantly greater pain intensity, H(1) = 6.77, *p* = 0.009, pain behaviour, H(1) = 7.42, *p* = 0.006 and pain interference, H(1) = 8.47, *p* = 0.002 than age-matched peers (Table 2).

### Subgroups with pain

Pain was experienced by participants in both groups. Of those who reported pain, 93% of the children with JIA and 90% of their peers had recurrent or persistent pain throughout the week. A Kruskal-Wallis test was used to compare pain intensity between the groups. Other variables were normally distributed and calculated by t-test. No significant difference was found between the groups in pain intensity, H(1) = 2.68, *p = 0.102* or pain behaviour, t(24) = 1.99, *p = 0.058*. However, the children with JIA and pain reported significantly more pain interference on daily activities than their healthy peers with pain t(24) = 2.43, *p = 0.023* (Table 3).

**Table 3:** The results from the PROMIS questionnaires from the subgroups of children who had experienced pain in the past 7 days

Questionnaire	JIA (***n*** = 16) mean ± SD	Control group (***n*** = 10) mean ± SD	***p***-value
PROMIS PAEDIATRIC PAIN INTENSITY (0-10)	5.25 ± 1.57	4.1 ± 1.66	0.102
PROMIS PAEDIATRIC PAIN BEHAVIOUR – SHORT FORM (T-score)	47.7 ± 3.71	44.3 ± 5.03	0.058
PROMIS PAEDIATRIC PAIN INTERFERENCE – SHORT FORM (T-score)	49.5 ± 5.97	43.4 ± 6.46	0.023

## Discussion

The findings of this study are that children with JIA in Iceland are more likely to experience pain than their peers, have a higher pain intensity and report a larger impact of pain on daily activities and behaviour. A significantly larger number of children with JIA than controls reported pain (57% compared to 27%). The proportion of children with pain in the latter group was similar to a study of healthy schoolchildren [8]. Half of the children with JIA and 25% of the children in the control group reported recurrent pain. A study from 2017 found that 25-50% of children in elementary schools experienced recurrent musculoskeletal pain, not caused by a disease, on average 2-3 weeks per year [29]. Reports of recurrent pain among children with JIA are quite variable in other studies. Schanberg et al. [21] demonstrated that most children with polyarticular juvenile arthritis who completed daily diaries for 2 months reported having pain for an average of 73% of the diary days. The JIA group in the current study was more heterogeneous in terms of type of arthritis, which may be reflected in a lower proportion of children reporting recurrent pain within the past week.

Most children in the control group reported pain in one body area. A systematic review by King et al. [8] reported that a high proportion of musculoskeletal pain in healthy children is caused by trauma and is located at one site. The results of the control group in this study were equivalent to that. The children with JIA had pain in significantly more body areas than their peers. They reported up to five painful body areas, indicating a more widespread pain, which demonstrates an important difference between the two groups. Bromberg et al. reported that the number of painful body areas at any given time in children with JIA was a predictor of activity limitations at that time, above and beyond the effects of pain intensity [6].

The group with JIA had an average pain intensity of 3/10 on the PROMIS numeric rating scale, which is significantly more than their peers who had average pain intensity of 1.17/10. The pain intensity of the children with JIA is consistent with results from Hanns et al. who reported average pain intensity of 3.3/10 on the visual analogue scale in their group [30].

Only six children (21.4%) in this study had appreciable joint inflammation meaning that 22 did not (78.6%). The average JADAS27 score was 2.9 ± 3.1, which is low disease activity [31]. Nonetheless, 57% reported pain. Bromberg et al. [6] reported the same; 66% of their study group reported episodes of pain in e-diary entries over a month but had an inactive disease. It must be kept in mind that pain perception in children with JIA is complex, and psychological- and environmental factors as well as central sensitisation can play a role in the pain experience [2, 13–18].

The children with JIA experienced a greater impact of pain on daily life and demonstrated more pain behaviour than their peers, which is consistent with previous research [18, 21]. The distribution of pain was greater among the children with JIA than their healthy peers which may explain more pain interference. Healthy children report pain episodes, but they expect the pain to be temporary [8]. Children with JIA, however, experience active and inactive episodes. They know that they can become better but also that the joint swelling and other symptoms can recur. Such ambiguity can impact children’s experience of pain and can interfere with daily life.

### Subgroups of children with pain

A closer look at the children who had experienced pain in the past week, showed that although the children with JIA and pain scored higher on the PROMIS scale than the healthy peers with pain (5.25/10 versus 4.1/10), the difference was non-significant. In terms of pain behaviour, the difference between the groups was non-significant, although it did approach significance. It should be noted that the sample is small which increases the risk for Type 2 error, and while the difference was non-significant, t was in the expected direction, i.e., children with JIA and pain displayed more pain behaviour than their healthy peers. Moreover, the children with JIA and pain reported significantly more pain interference than their healthy peers with pain. The authors of this study are not aware of other studies where pain intensity, pain interference and pain behaviour were compared between groups of children with JIA who reported pain and a group of children from the general population who reported pain. Comparison of these two subgroups in this study indicated different impacts of pain between the two groups of children experiencing pain. However, the subgroups were small but our study’s results might indicate the need for further exploration of the impact of pain on the two groups of children experiencing pain.

### Strengths and limitations

The study results should be interpreted with several limitations in mind. This was a cross-sectional observational study; therefore, no causal inferences can be made and there may have been unmeasured confounding factors. The data were based on self-reports, which are inherently subjective and rooted in individual personality, outlook, and context. However, subjective measures do provide us with important information about the way the children perceive their pain independent of its cause. While the use of an objective performance-based measurement including a set of tasks would have given the study an added value few such measurements have been developed specifically for children with juvenile arthritis. No measurement tool includes tasks of a wide spectrum of activities, like walking, running, transferring, stair climbing, getting dressed, writing and other fine motor activities.

The study may be prone to non-response bias, in that participants may potentially have more interest in health, JIA, and physical activity than non-participants, as these topics were all within the scope of a large research project that this study was a part of. Due to the small population in Iceland, there is a rather small number of children with JIA, which increases the risk for type 2 error and makes generalisation of the results to other groups difficult. It should be kept in mind, that the sample only included families who lived within a 100 km radius of the capital region where the only Pediatric Rheumatology Outpatient Clinic is situated. This leaves out 10 children with JIA who belong to the clinic but must travel far for control visits and treatment. This could have biased the results in such a small study. In addition, the small sample makes it impossible to look at differences among those with active and inactive disease, age groups or different subtypes of JIA.

The strengths of the study include the use of the three PROMIS scales, which are short and standardised questionnaires with established psychometric properties. The questions are clear and user-friendly. The use of the questionnaires had the benefit of being time- and cost-efficient and easy to administer. All the questions were on pain and the impact of pain during the past 7 days. When children experience episodes of joint inflammation and pain, it is more valuable to ask them about pain experienced over a week, rather than on the 1 day when they respond to the survey. The response rate of the children with JIA was 58%, without reimbursement. There is no consensus on what a good response rate is in a survey but generally responses of 30% or more is considered high [32]. So, while the study’s sample includes few children, it represents a good portion of the population of 8–17-year-old children with JIA in Iceland.

The results give an overview of pain, pain behaviour and pain impact on children with JIA in Iceland. The control group was similar in age, sex, height, weight, and body mass index (BMI), which avoids biases due to differences between the groups. Finally, this is the first study of pain and impact of pain on children with JIA in Iceland.

## Conclusions

The results indicate that the number of pain areas, frequency and intensity of pain was different between of children with JIA and age-matched peers. Moreover, children with JIA demonstrated significantly more pain behaviour and pain interference on the daily lives, than controls. Further research investigating the role of pain management on functioning, measured with various measurement tools, in children with juvenile idiopathic arthritis is needed. Moreover, further exploration of the impact of different aspects of the pain experience on children with JIA is needed. A larger study over a longer period where the data is collected with daily diaries of pain and its effects on daily activities could examine differences between subtypes of JIA and between children with active and inactive disease.

## Data Availability

The datasets used and/or analysed during the current study are available from the corresponding author on reasonable request.

## Abbreviations

JIA: Juvenile idiopathic arthritis
PROMIS: Patient-Reported Outcome Measurement Information System
